# Predictive quality assurance of a linear accelerator based on the machine performance check application using statistical process control and ARIMA forecast modeling

**DOI:** 10.1002/acm2.12917

**Published:** 2020-06-15

**Authors:** Wayo Puyati, Amnach Khawne, Michael Barnes, Benjamin Zwan, Peter Greer, Todsaporn Fuangrod

**Affiliations:** ^1^ Department of Computer Engineering Faculty of Engineering King Mongkut’s Institute of Technology Ladkrabang Bangkok 10520 Thailand; ^2^ Department of Mathematics Statistics and Computer Faculty of Science Ubon Ratchathani University Ubon Ratchathani 34190 Thailand; ^3^ Department of Radiation Oncology Calvary Mater Hospital Newcastle NSW 2298 Australia; ^4^ School of Mathematical and Physical Sciences University of Newcastle Newcastle NSW 2308 Australia; ^5^ Central Coast Cancer Centre Gosford Hospital Gosford NSW 2250 Australia; ^6^ Faculty of Medicine and Public Health HRH Princess Chulabhorn College of Medical Science Chulabhorn Royal Academy Bangkok 10210 Thailand

**Keywords:** autoregressive integrated moving average forecast modeling, machine performance check, predictive quality assurance, statistical process control

## Abstract

**Purpose:**

A predictive linac quality assurance system based on the output of the Machine Performance Check (MPC) application was developed using statistical process control and autoregressive integrated moving average forecast modeling. The aim of this study is to demonstrate the feasibility of predictive quality assurance based on MPC tests that allow proactive preventative maintenance procedures to be carried out to better ensure optimal linac performance and minimize downtime.

**Method and Materials:**

Daily MPC data were acquired for a total of 490 measurements. The initial 85% of data were used in prediction model learning with the autoregressive integrated moving average technique and in calculating upper and lower control limits for statistical process control analysis. The remaining 15% of data were used in testing the accuracy of the predictions of the proposed system. Two types of prediction were studied, namely, one‐step‐ahead values for predicting the next day's quality assurance results and six‐step‐ahead values for predicting up to a week ahead. Results that fall within the upper and lower control limits indicate a normal stage of machine performance, while the tolerance, determined from AAPM TG‐142, is the clinically required performance. The gap between the control limits and the clinical tolerances (as the warning stage) provides a window of opportunity for rectifying linac performance issues before they become clinically significant. The accuracy of the predictive model was tested using the root‐mean‐square error, absolute error, and average accuracy rate for all MPC test parameters.

**Results:**

The accuracy of the predictive model is considered high (average root‐mean‐square error and absolute error for all parameters of less than 0.05). The average accuracy rate for indicating the normal/warning stages was higher than 85.00%.

**Conclusion:**

Predictive quality assurance with the MPC will allow preventative maintenance, which could lead to improved linac performance and a reduction in unscheduled linac downtime.

## 1 INTRODUCTION

Daily quality assurance (QA) testing of linear accelerators (linacs) is standard practice for ensuring the safe and accurate delivery of radiotherapy treatments. The American Association of Physicists in Medicine (AAPM) has published recommendations on daily linac QA procedures with tolerances.^1^, ^2^, ^3^ However, the test equipment and exact methodology are non‐standardized and remedial action is often only reactively performed once tolerances have been breached.

With the TrueBeam 2.0 platform, Varian (Varian Medical Systems, Palo Alto, CA, USA) released the Machine Performance Check (MPC) application, which is now also available on the Varian Halcyon linac. MPC is a fully integrated image‐based tool for assessing the performance of linac critical functions. MPC tests are divided into two categories. First, beam constancy checks use a single megavolt image per beam energy without a phantom in place to assess the output, beam center, and uniformity constancy against a user‐defined baseline. Second, geometric tests use a series of kilovoltage and 6 MV beam images of the IsoCal phantom situated in a specific bracket on the couch top to assess the radiation isocenter size, coincidence of megavolt and kilovolt isocenters, accuracy of collimator and gantry angles, accuracy of jaw and multileaf collimator (MLC) leaf positions, and accuracy of the couch positioning including pitch and roll. All measurements are highly automated, and the user is simply required to set up the IsoCal phantom and bracket on the treatment couch at position H2 and to beam‐on for each required energy. For the geometric tests, the system makes all required motions automatically and beams‐on when all is in position. Images are automatically analyzed at the linac console. Beam constancy check results are presented relative to the baseline, whereas geometric tests are self‐referenced. Functionality for presenting trends in results is also embedded in the MPC module and data can be exported in.csv format. The MPC application has now been evaluated by multiple authors as a daily linac QA tool.^4^, ^5^, ^6^, ^7^, ^8^

Statistical process control (SPC) is a statistical method for detecting the defects of a process and was first presented by Shewhart.^9^ SPC has become a standard method of quality control. In SPC, continual observations are used to calculate a control chart, which includes a maximum control limit and minimum control limit that define a quality level. The control chart is often used in monitoring a process and detecting failure states at a point of measurement. Binny et al. applied SPC to analyze the QA output variation in helical and static output for periods of up to 4 yr in an effort to improve helical tomotherapy QA.^10^ Meanwhile, López‐Tarjuelo et al. adopted SPC in the daily quality control of linac electron beams.^11^ Fuangrod et al. applied SPC in constructing a clinically significant threshold of a real‐time treatment verification system.^12^ Recently, SPC has been used with MPC data in another study by Binny et al.^13^ In that study, SPC analysis was conducted for MPC data across six TrueBeam linacs for 12 months in an attempt to determine MPC tolerances. However, the study did not attempt to forecast MPC results to be used for predictive QA.

The concept of predictive QA that allows preemptive maintenance based on SPC analysis has been studied for radiotherapy linacs.^14^, ^15^, ^16^, ^17^ Predictive QA testing would allow radiotherapy departments to be proactive in their maintenance by remedying faults before clinical tolerances are breached. In theory, this should provide for improved linac performance consistency and reduced unscheduled linac downtime that can be disruptive to departmental workflow. Previous predictive QA studies have either been based on readouts from the linac itself^15^, ^16^, ^17^ and hence lack independence or have been based on film measurements,^14^ which are impractical on a daily basis.

The present study presents a framework of predictive linac QA based on MPC data. Control limits were determined from the SPC analysis of MPC data over the long term and used to determine the standard linac performance. When this performance is within clinical tolerances, there is a window of opportunity to rectify the problem before it becomes clinically significant. Such remedial action can be scheduled out of standard treatment hours to avert disruption to the clinical workflow. The forecasting of QA results provides a measure of how long this window of opportunity is.

## 2 MATERIALS AND METHODS

### 2.A System overview

**Fig. 1:**
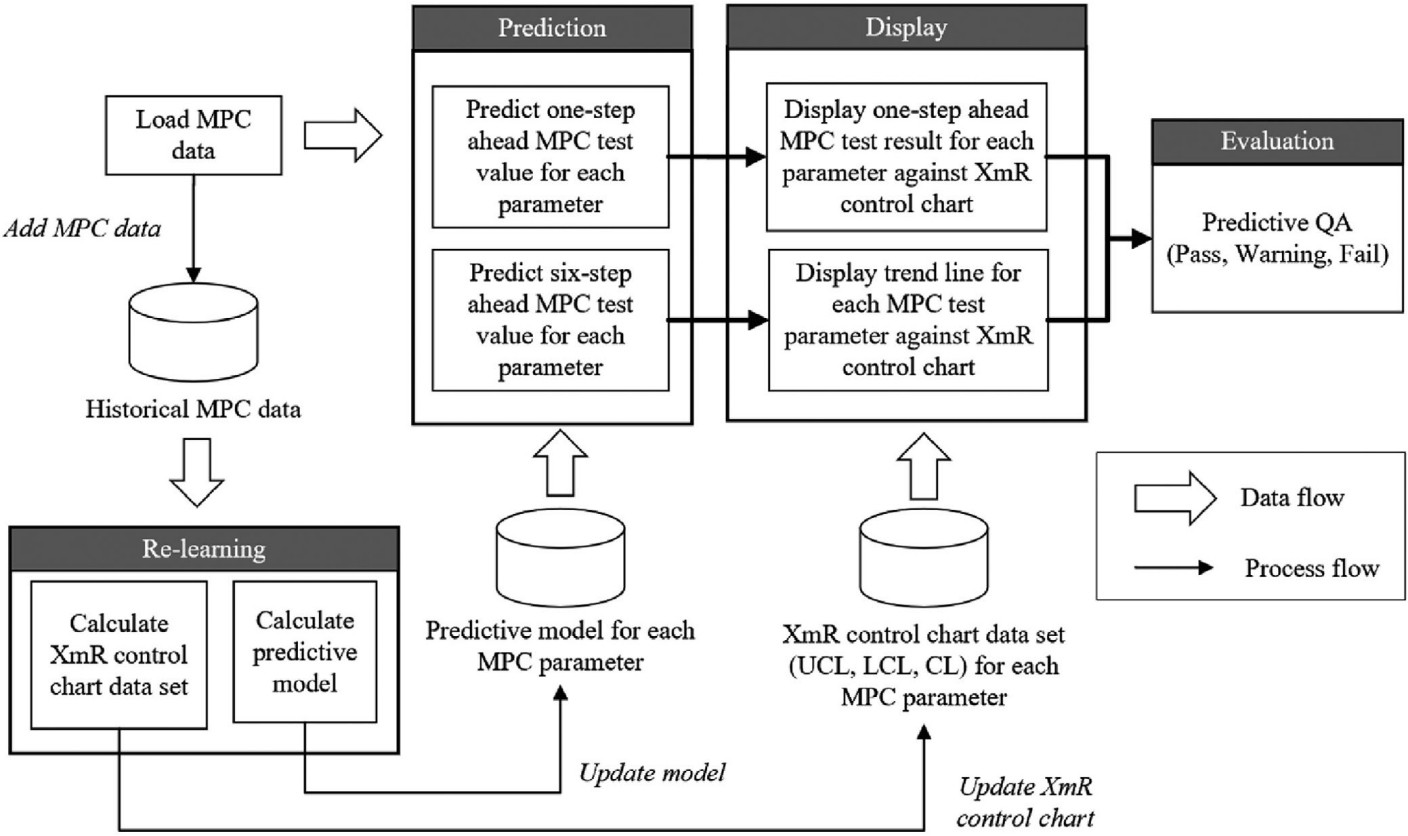
System overview.

Figure 1 presents the proposed predictive QA system, which can be divided into the four steps of prediction, display, evaluation, and relearning. Daily MPC test data are prepared and loaded into the system. The system predicts both one‐step‐ahead and six‐step‐ahead MPC test results using a predictive model, which has been learnt from historical MPC data. In the display step, the predictive MPC data for each parameter are displayed on the constructed control chart which shows the upper control limit (UCL), lower control limit (LCL), central line (CL), and tolerance levels. The tolerance levels are defined by AAPM TG‐142 recommendations.^1^ However, some MPC tests are not required by TG‐142 daily QA, the standard MPC thresholds were used instead. The trend of predictive data is also plotted from the predictive six‐step‐ahead MPC test result for each parameter. The system was developed and implemented using in‐house software development using MATLAB version 2019a (Mathworks, Natick, MA, USA).

In the evaluation step, the predictive MPC test results are compared with the UCL and LCL and the system generates a warning flag if the control limits are breached. This flag escalates from warning to fail if the result exceeds the TG‐142 tolerance. Finally, the MPC test results are added to the historical MPC data for relearning and recalculating the UCL and LCL. The system therefore self‐adjusts to reflect the current linac uncertainty and behavior.

### 2.B MPC tests and data collection

There are 29 MPC tests that can be grouped into four categories, namely, isocenter, collimation, gantry, and couch tests. In collimation, there are four MLC tests — denoted mean offset leaf‐bank A and B and max offset leaf‐bank A and B — and the system also provides results for individual MLC leaves from leaf number 2 to 59 for both banks A and B. There are thus a total of 141 MPC test parameters (25 MPC tests + 116 individual MLC leaves). For the predictive model and SPC development, historical MPC data were taken from daily MPC tests from a single Varian TrueBeam Stx linac with HD MLC, 6MV energy, and As 1200 EPID over 490 days. The data were divided into two data sets. The first 85% of data (416 days) were used in constructing the SPC control chart and calculating the predictive model for each MPC test. The remaining 15% of data (74 days) were compared to the predictive model results to validate its accuracy and evaluate the system performance.

### 2.C Control chart construction

The control chart ^9^ and UCL and LCL are constructed according to

(1) $$UCL=\bar{x}+E\cdot \bar{\mathrm{MR}}\;LCL=\bar{x}-E\cdot \bar{\mathrm{MR}}\;CL=\bar{x}$$

where $\bar{x}$ is the average of m data observations, $\bar{\mathrm{MR}}$ is an average of the moving range $\left(\bar{\mathrm{MR}}=\frac{\sum \left|x_{i}-x_{i-1}\right|}{m-1}\right)$, and E is 2.66.^18^ *UCL*, *LCL*, and *CL* are, respectively, the UCL, LCL, and CL.

### 2.D Predictive model calculation: autoregressive integrated moving average

The autoregressive integrated moving average (ARIMA) model is a statistical predictive model whose fitting allows future prediction of the time series.^9^ The ARIMA methodology includes an autoregressive term (AR), which is the weighted sum of recent differenced values, moving average term (MA), which is the weighted sum of the forecasting error, and integrated term, which is the degree of differencing for nonstationary elimination, if necessary. The general form of ARIMA (p,d,q) is as follows:

(2) $${\hat{z}}_{t}=\mu +\left(\phi_{1}z_{t-1}+\ldots +\phi_{p}z_{t-p}\right)-\left(\vartheta_{1}a_{t-1}+\ldots +\vartheta_{q}a_{t-q}\right),$$

where ${\hat{z}}_{t}$ is the predictive value at t, $z_{t}$ denotes differenced values in the time series at t, μ is a constant value, $\phi_{i}$ denotes the weights of differenced values of AR ( i=1,2,…,p), $a_{t}$ denotes the orders of predicted error, $\vartheta_{j}$ denotes the weights of predicted error ( j=1,2,…,q), p is the order of AR, q is the order of the MA, and d is the order of differencing in nonstationary elimination.

Maximum likelihood estimation is adopted to estimate the parameter and error terms of the ARIMA model. The Akaike information criterion^20^ is used to select the optimal model, which is the model that best fits the recorded time‐series data. The ARIMA model is also used to predict the n‐step‐ahead value, which can be used to represent the future trend of the time‐series data.

### 2.E Predictive model and system evaluation

The accuracy of the predictive model was evaluated by comparing the ARIMA predicted results with the actual results in the final 15% of the MPC data. Model accuracy was assessed using the root‐mean‐square error (RMSE) and absolute error (MAE).

(3) $$RMSE=\sqrt{\sum_{i=1}^{n}\frac{{\left({\hat{y}}_{i}-y_{i}\right)}^{2}}{n}}$$

(4) $$MSE=\frac{1}{n}\sum_{i=1}^{n}{\left({\hat{y}}_{i}-y_{i}\right)}^{2}$$

when ${\hat{y}}_{i}$ is the predicted value, $y_{i}$ is the actual value of state i, and n is the number of observations. RMSE and MSE are considered as the standard error measurements of model in predicting quantitative data. In addition, the model can also be evaluated by comparing the predicted results against the control chart (pass, fail, or warning status). The accuracy of the model in this sense can be calculated using the average accuracy:

(5) $$\text{Average}\;\text{accuracy}=\frac{1}{l}\sum_{i=1}^{l}\left(tp_{i}+tn_{i}\right)/\left(tp_{i}+tn_{i}+fp_{i}+fn_{i}\right)$$

The average accuracy rate represents the average overall effectiveness of prediction. Let $tp_{i}$ be the number of true positives of state i, which is the number of correct predictions of state i. Let $tn_{i}$ be number of the true negatives of state i, which is the number of correct predictions of nonstate i. Let $fp_{i}$ be the number of the false positives of state i, which is the number of incorrect predictions of state i. Let $fn_{i}$ be the number of false negatives of state i, which is the number of incorrect predictions of nonstate i.

## 3 RESULTS

### 3.A SPC analysis

**Fig. 2:**
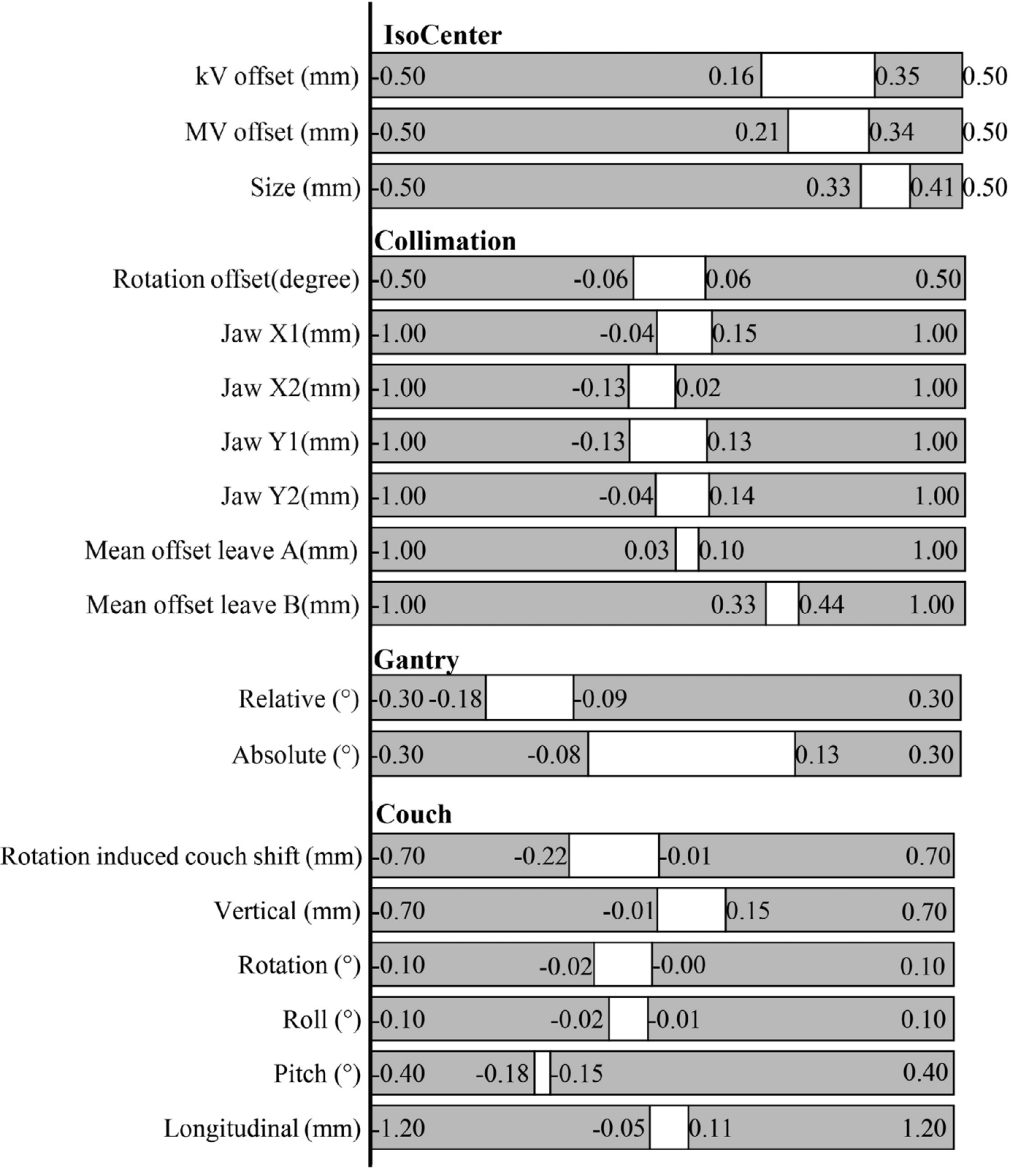
Results of the derived LCL and UCL of the MPC (isocenter, collimation, gantry, and couch) against the TG 142 tolerance.

**Fig. 3:**
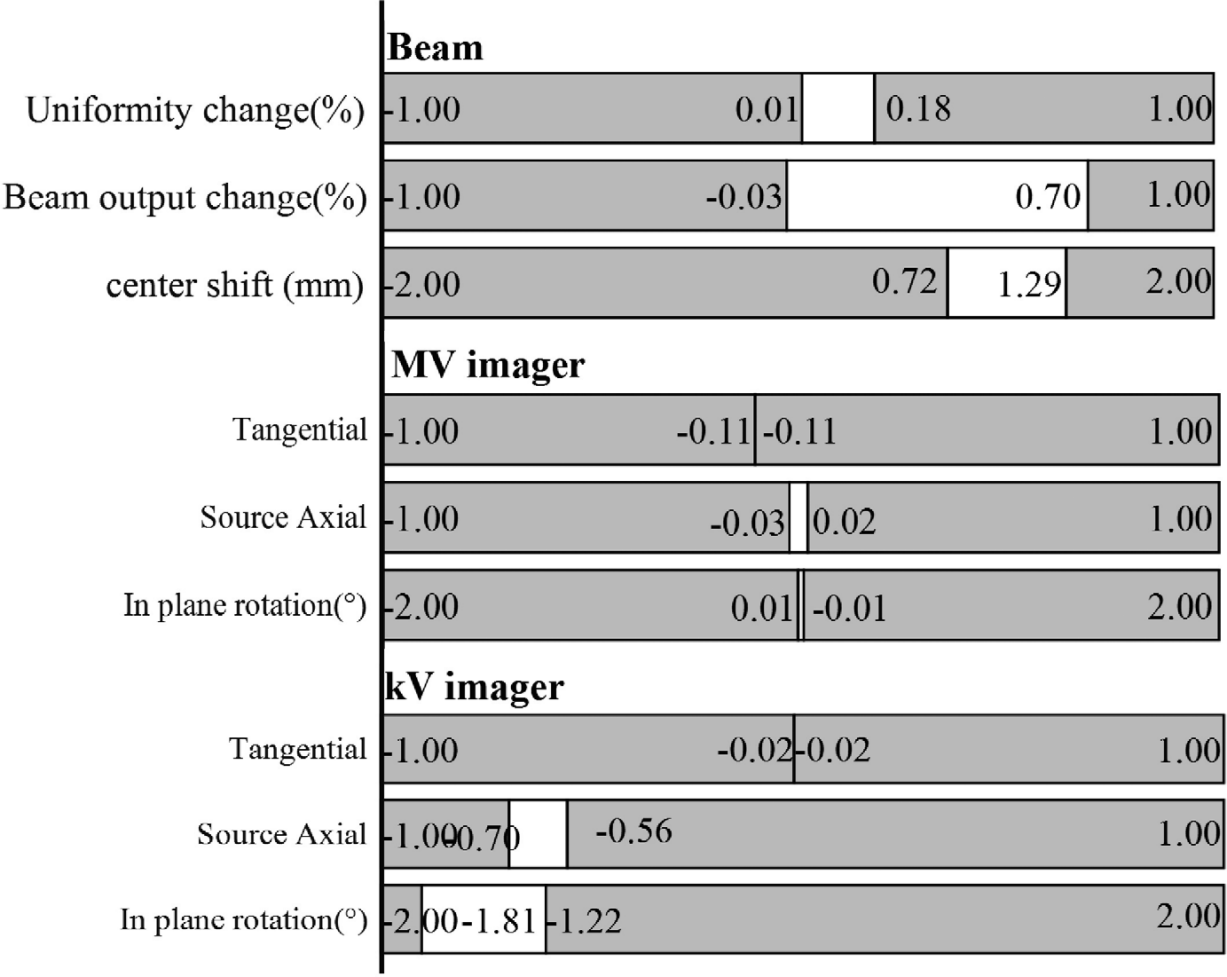
Results of the derived LCL and UCL of the MPC (beam, MV imager, and kV imager) against the TG‐142 tolerance.

Figures 2 and 3 present the UCL and LCL for each MPC test. In the figures, a white area represents the normal stage level (ranging from the UCL to LCL) and a grey area is a warning stage level (ranging from the UCL/LCL to the tolerance).

### 3.B Predictive QA system

**Fig. 4:**
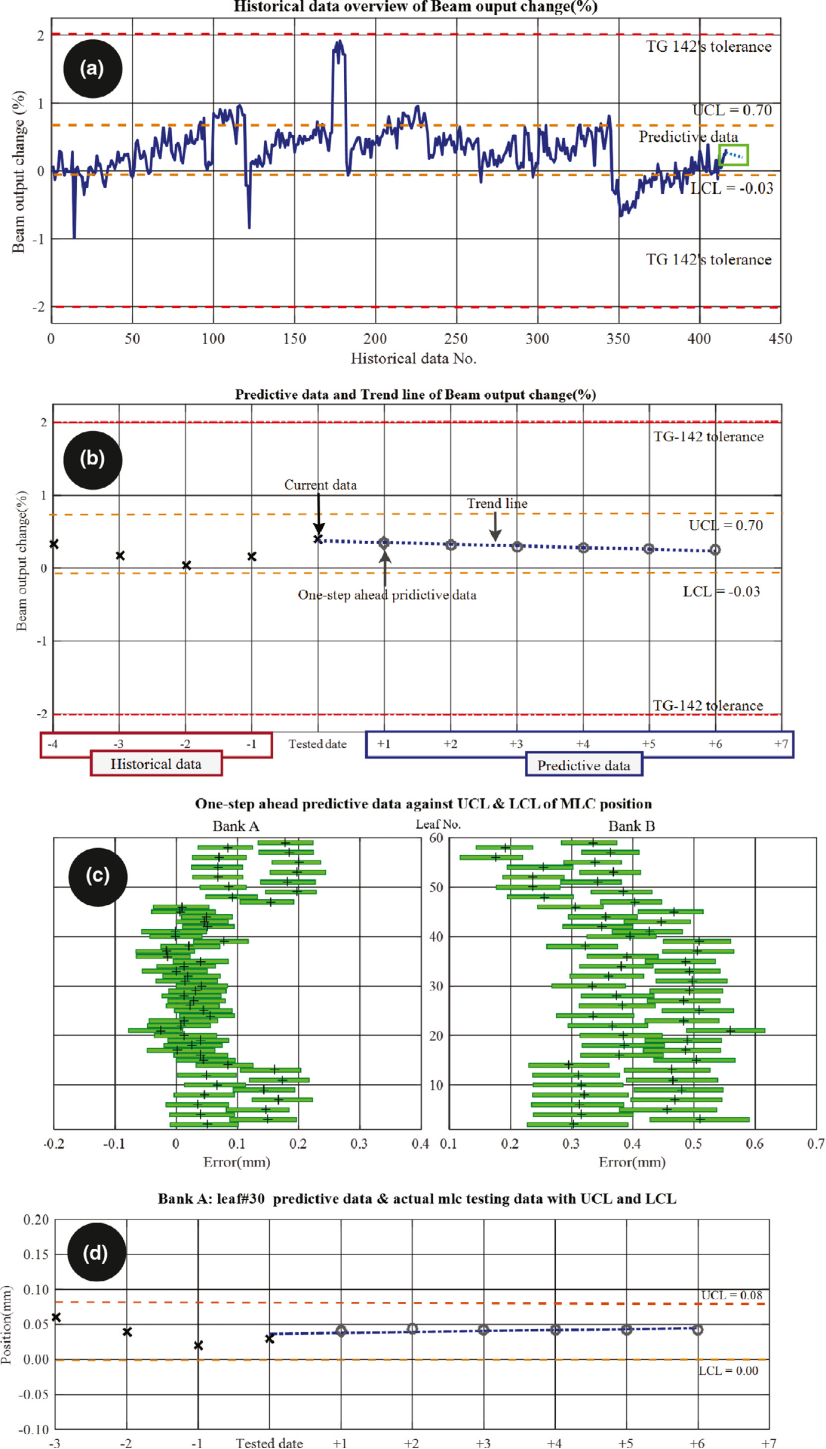
(a) Historical overview of the beam output change (%); (b) sample of one‐step‐ahead predictive data, six‐step‐ahead predictive values, and trend of the beam output change; (c) overview of the one‐step‐ahead prediction of the MLC against XmR; and (d) a sample comparison of the current check and one‐step‐ahead prediction of the MLC with XmR and the trend line for leaf No. 30.

**Fig. 5:**
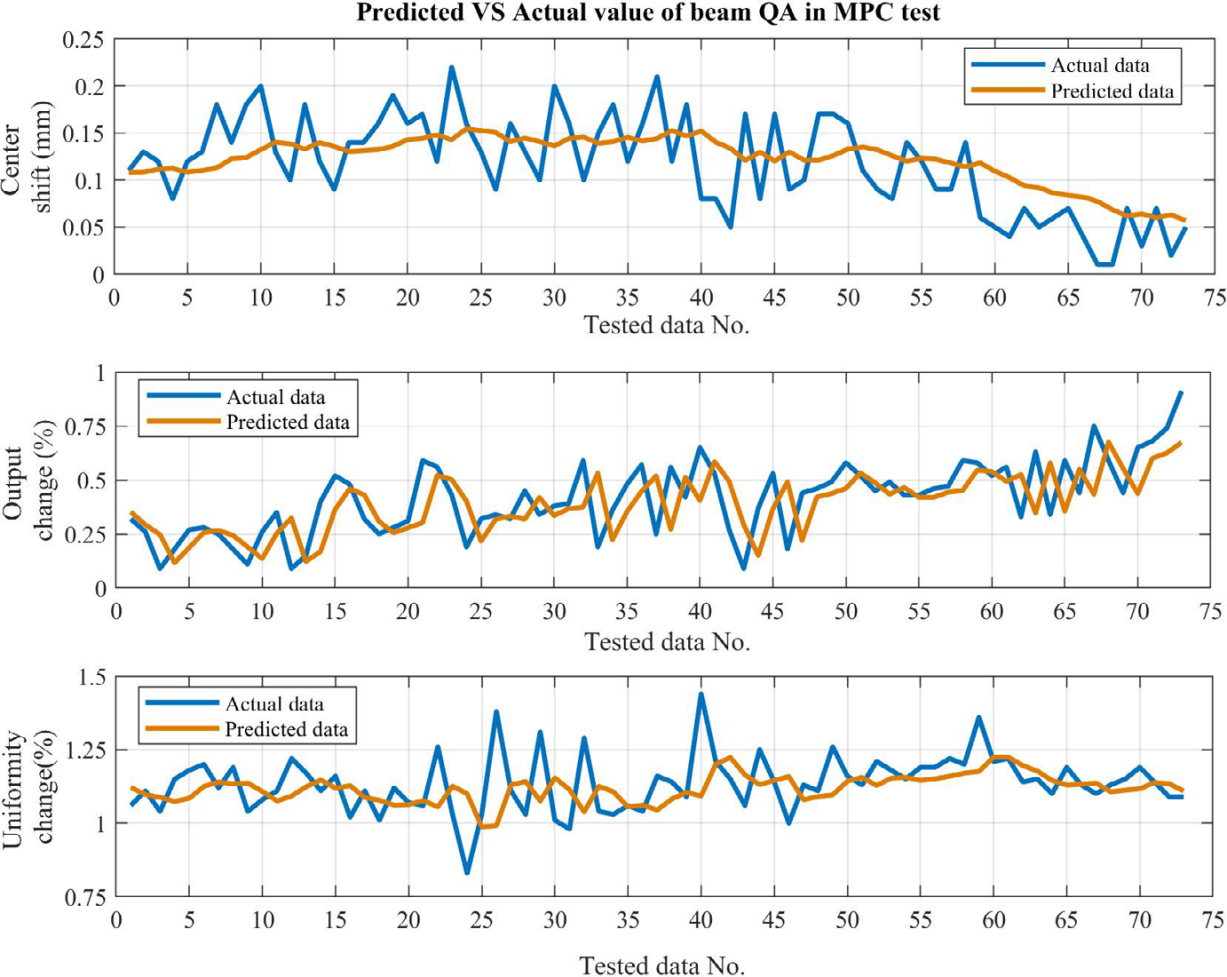
Comparison of predicted and actual beam QA data including center shift, output change, and uniformity change.

**Fig. 6:**
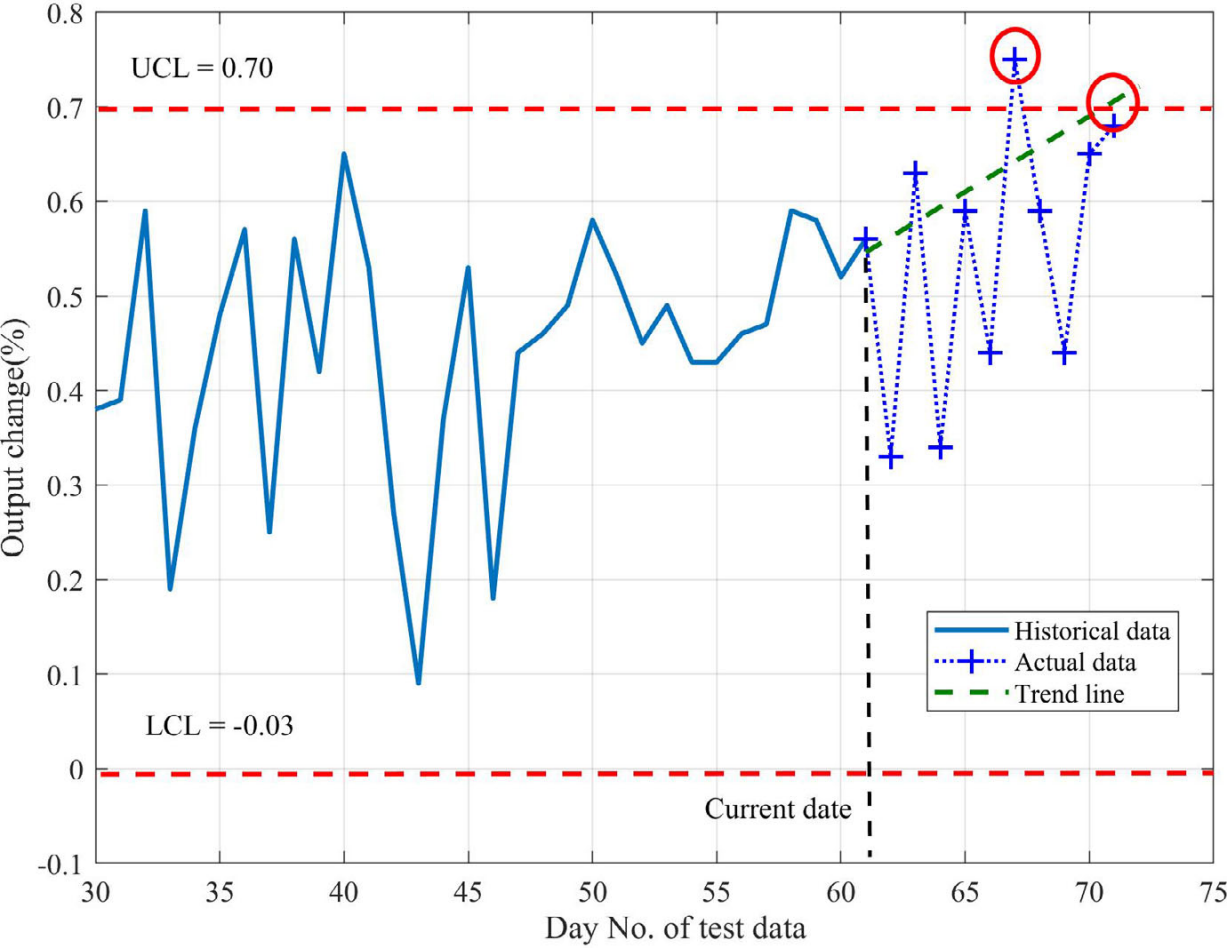
Example of trend line to detect the output change exceeded the UCL that demonstrates the system is able to flag the warning stage before it occurs.

Figure 4 shows an example of predicted QA data. Figure 4(a) and 4(b) present the MPC test for the beam output change. The one‐step‐ahead predictive value is calculated as well as its weekly trend line (from six‐step‐ahead predictive values). All predictive values are within the SPC control limits and the trend is that the beam output slightly drops over the week but remains at normal‐stage levels. Figure 4(c) presents prediction results of individual MLC leaf tests, showing that the one‐step‐ahead result remains located at normal‐stage levels for all leaves. Figure 4(d) presents the six‐step‐ahead prediction for MLC leaf #30. The prediction indicates that the leaf remains between the UCL and LCL. Figure 5 presents the comparison between predicted and actual of beam center shift, beam output change, and beam uniformity change for all tested data. It found that the beam output change had the systematic offset in predicted value. Figure 6 demonstrated how the system predicts that the data will exceed the warning level (UCL). Figures are not presented for all MPC parameters for readability purposes.

### 3.C Predictive model performance

The performance of the ARIMA model in predicting daily MPC results is presented in Table 1. The majority of tests make accurate predictions with an RMSE less than or equal to 0.05. Exceptions are the jaw collimation, gantry relative, kilovolt imager tangential, beam output change, and beam uniformity change, with the worst case being the output measured at 0.14 RMSE. A lower RMSE corresponds to the predictive model better correlating with the actual MPC test results. A similar pattern of evaluation results was observed with the MAE metric in that most of the results are less than or equal to 0.05. Figure 5 presents the results for individual MLC leaves. The results show that the high numbered leaves are predicted better than the low numbered leaves for Bank A, while the prediction is more consistent across the bank for bank B. The reason for these results is unclear.

**Table 1:** Results of the predictive model evaluation and accuracy of system output.

MPC test		RMSE	MAE	Average accuracy rate (%)
**Categories**	**Test**			
Isocenter	Size (mm)	0.02	0.01	97.80
	MV imager projection offset (mm)	0.03	0.02	79.12
	kV imager projection offset (mm)	0.04	0.03	61.54
Collimation	Maximal offset leaves A (mm)	0.02	0.02	100.00
	Maximal offset leaves B (mm)	0.03	0.03	98.97
	Mean offset leaves A (mm)	0.02	0.02	98.97
	Mean offset leaves B (mm)	0.03	0.02	98.97
	Individual MLC leaf A (mm)	0.03	0.02	98.56
	Individual MLC leaf B (mm)	0.03	0.02	98.13
	Jaw X1 (mm)	0.10	0.06	47.42
	Jaw X2 (mm)	0.13	0.05	95.88
	Jaw Y1 (mm)	0.09	0.07	64.21
	Jaw Y2 (mm)	0.06	0.05	79.17
	Rotation offset (°)	0.02	0.02	100.00
Gantry	Absolute (°)	0.02	0.01	92.86
	Relative (°)	0.06	0.06	100.00
Couch	Lateral (mm)	0.04	0.04	100.00
	Longitudinal (mm)	0.03	0.02	64.86
	Pitch (°)	0.01	0.01	98.63
	Roll (°)	0.01	0.00	95.38
	Rotation (°)	0.01	0.01	48.10
	Vertical (mm)	0.05	0.04	91.78
	Rotation‐induced couch shift (mm)	0.02	0.02	100.00
Kilovolt imager	In‐plane rotation (°)	0.00	0.00	100.00
	Source axial	0.04	0.03	95.60
	Tangential	0.12	0.10	98.90
Megavolt imager	In‐plane rotation (°)	0.00	0.00	100.00
	Source Axial	0.01	0.01	60.44
	Tangential	0.01	0.01	100.00
Beam	Center shift (mm)	0.05	0.04	89.52
	Beam output change (%)	0.14	0.12	94.29
	Uniformity change (%)	0.10	0.07	93.33

The average accuracy rate was applied to assess the ability of the models to accurately flag the warning stage. The fourth column in Table I shows the results of the average accuracy rate of the one‐step‐ahead prediction. It is seen that most of the average accuracy rates for all tests were higher than 85.00%. Exceptions are the tests denoted megavolt and kilovolt imager projection offset, Jaw X1, Jaw Y1 & Y2, couch longitudinal and rotation, and megavolt imager source axial. It can be noted that QA test with minor variability or drift will result in tight UCL and LCL so that sensitivity is maintained.

**Fig. 7:**
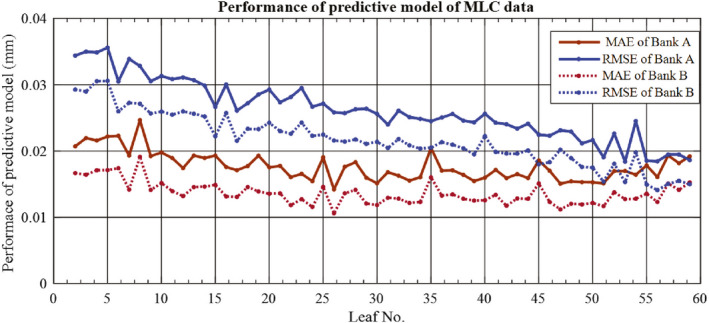
Performance of the predictive model for individual leaves evaluated using the MAE and RMSE.

**Fig. 8:**
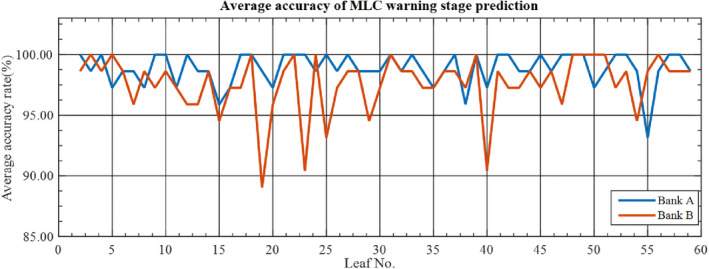
Evaluation of the warning stage prediction using the average accuracy for an individual leaf of the MLC.

Figures 7 and 8 present the results of the average accuracy for individual MLC leaves. The average accuracy is more than 90.00% for all leaves of both banks with the exception of leaf number 19 of bank B.

## 4 DISCUSSION

MPC is advantageous for providing predictive QA data as MPC:

1. provides a measurement that is independent of the linac parameters being tested.
2. is quick and easy to perform and thus suitable for daily measurements, producing large, high‐frequency datasets for predictive analysis.
3. is largely free of user variability.
4. assesses a large number of required linac parameters, meaning that other test devices are not required.
5. generates digital data that are integrated into the linac system, making it simple for Varian to transmit results to a central database via its Smart Connect functionality.
6. provides a standardized method for daily linac QA, meaning that the performances of all linacs running the MPC can be compared. This has the potential for individual linacs with outlying performance to be identified and to provide feedback data to Varian for improvement of the linac design and construction.

In this study, the ARIMA forecasting model was applied to predict both the next‐day (one‐step‐ahead) and next‐week (six‐step‐ahead) MPC QA results. SPC control charts were constructed to determine the upper and lower bounds of standard machine performance. For all parameters tested, these bounds were found to be within clinical tolerances (defined as TG‐142 tolerances in this study), providing a window of opportunity in which a performance issue could be rectified before it became clinically significant.

For the MLC results of [Fig. 4(c)], individual models were generated for the leave banks and individual leaves. Since the leaf bank results are maximum and mean of all of the individual leaves, then it is expected that the model for each individual leaf would be more precise than for the whole bank. This is demonstrated in the data.

The accuracy required for prediction in the clinical setting is likely not high. Clinical physicists would most likely simply need a rough indication of how long they have before a failed test is expected so that they can assign urgency to the problem being addressed and organize remediation. For a TG‐142 tolerance fail, the decision may well be to remove the linac from clinical use until the failure has been investigated, but for results within the warning stage between control limits and clinical tolerance, the investigation would likely be delayed until outside of normal treatment hours to avoid disruption to the clinical schedule. In these cases, it is likely that investigation will be made at either the end of the clinical day or on the weekend. In these cases, one‐step‐ahead and six‐step‐ahead prediction is appropriate.

The testing in the present study found that the majority of the one‐step‐ahead predictions were accurate (RMSE and MAE << 0.10) and the majority of average accuracy results were greater than 85.00%. The worst predictive performance was for the beam output change. The beam output change has high variability over time and is recorded as a nonstationary time‐series dataset, which affects the accuracy of the predictive model. Resolution of this problem may require a different predictive model. Examples of possible models include the EWMA model, recurrent neural networks,^21^ and artificial neural networks,^22^ which could be investigated in future work.

An additional weakness of the ARIMA model is that the data require cleaning to eliminate poor‐quality data, such as user errors. The ARIMA model is sensitive to all values in the learning process and learning from poor‐quality data will lead to poor prediction. Moreover, the learning should continually progress by taking new measurements into account.

A possible source of error in the prediction model could be caused by EPID detector drift distorting the MPC results. To mitigate this source of uncertainty, throughout the study the EPID dark‐field calibration was updated monthly and pixel defect map annually or as dead pixels were identified. It is noted that MPC is independent of the flood field calibration. EPID response constancy has also been extensively studied in the literature. The short‐ and long‐term dose–response reproducibility of amorphous Silicon EPIDs has been found to be consistently within 1.0 % and 0.5 %, respectively, for all models once linac output variation from nominal had been accounted.^23^, ^24^, ^25^, ^26^, ^27^ A study by King et al^28^ pertained specifically to the long‐term pixel stability of Varian aS500 EPID panels. In King's study it was found that over a 3‐year period mean pixel variations were between 0.29 and 0.6 % and that more than 99% of all pixels showed variations less than 1 %. Also the MPC studies of ^5–7^ provided comparison of MPC tests against independent measurements over the medium term with good agreement generally observed. An exception to this was for the output parameter for which Barnes et al detected a divergence of MPC output vs. independent measures. Barnes et al suggest that this can be mitigated via regular intercomparison of MPC output with ion‐chamber readings and rebaselining MPC if a 1 % difference is detected. Such a measure, along with regular routine EPID calibration and QA, is recommended if the forecasting methodology of this study was implanted clinically.

Maintenance and recalibration events are also a potential source of sudden systematic change in MPC results as demonstrated by Barnes et al.^29^ These events were not accounted for in this study. Such systematic changes will influence the prediction model and hence at such events the prediction model input data should be reassessed and model training data collection potentially restarted.

Due to the nature of prediction models being based on large learning‐phase datasets the prediction models are not designed to detect large sudden one‐off jumps in data such as might be expected with a linac component failure. Linac interlocks are still required to mitigate treatment delivery errors from such events as well as routine retrospective QA. Predictive QA is more suited to detecting and forecasting gradual drifts and failures that repeat at regular intervals.

The results of the present study suggest that the approach of predictive QA based on MPC data is feasible, but additional data on more linacs are required and the method needs to be tested further in terms of sensitivity for the system to be clinically useable. Such study is proposed as future work.

## 5 CONCLUSION

The concept of linac predictive QA with the MPC using SPC and the ARIMA forecast model was demonstrated and its accuracy and performance evaluated. A window of opportunity between SPC control limits and clinical tolerances based on TG‐142 was demonstrated, suggesting that the MPC is an appropriate tool for predictive QA. The concept can be developed further with a greater number of linacs, sensitivity testing, and the evaluation of other predictive model techniques. Such testing thus has the potential to reduce the linac unscheduled downtime and allow linac performance parameters to be controlled within tolerances tighter than those typically applied in the clinic.

## CONFLICT OF INTEREST

The authors have no conflict of interest to disclose.
